# Identification of a Linear B Cell Epitope on p54 of African Swine Fever Virus Using Nanobodies as a Novel Tool

**DOI:** 10.1128/spectrum.03362-22

**Published:** 2023-05-16

**Authors:** Huijun Zhao, Gaijie Wang, Haoxin Dong, Shuya Wu, Yongkun Du, Bo Wan, Pengchao Ji, Yanan Wu, Dawei Jiang, Guoqing Zhuang, Hong Duan, Gaiping Zhang, Angke Zhang

**Affiliations:** a College of Veterinary Medicine, Henan Agricultural University, Zhengzhou, Henan, China; b International Joint Research Center of National Animal Immunology, College of Veterinary Medicine, Henan Agricultural University, Zhengzhou, Henan, China; c Henan Engineering Laboratory of Animal Biological Products, College of Veterinary Medicine, Henan Agricultural University, Zhengzhou, Henan, China; d Longhu Modern Immunology Laboratory, Zhengzhou, Henan, China; Wright State University

**Keywords:** ASFV, nanobodies, B cell epitopes

## Abstract

African swine fever (ASF) has received great attention from the swine industry due to the pandemic and the lack of vaccines or effective treatments. In the present study, 13 African swine fever virus (ASFV) p54-specific nanobodies (Nbs) were successfully screened based on Bactrian camel immunization of p54 protein and phage display technology, and their reactivity with the p54 C-terminal domain (p54-CTD) was determined; however, only Nb8-horseradish peroxidase (Nb8-HRP) exhibited the best reactivity. Immunoperoxidase monolayer assay (IPMA) and immunofluorescence assay (IFA) results indicated that Nb8-HRP specifically reacted with ASFV-infected cells. Then, the possible epitopes of p54 were identified using Nb8-HRP. The results showed that Nb8-HRP could recognize p54-CTD truncated mutant p54-T1. Then, 6 overlapping peptides covering p54-T1 were synthesized to determine the possible epitopes. Dot blot and peptide-based enzyme-linked immunosorbent assay (ELISA) results suggested that one novel minimal linear B cell epitope, ^76^QQWVEV^81^, which had never been reported before, was identified. Alanine-scanning mutagenesis revealed that ^76^QQWV^79^ was the core binding site for Nb8. Epitope ^76^QQWVEV^81^ was highly conserved among genotype II ASFV strains and could react with inactivated ASFV antibody-positive serum from naturally infected pigs, indicating that it was a natural linear B cell epitope. These findings provide valuable insights for vaccine design and p54 as an effective diagnostic tool.

**IMPORTANCE** The ASFV p54 protein plays an important role in inducing neutralization antibodies *in vivo* after viral infection and is often used as a candidate protein for subunit vaccine development. The full understanding of the p54 protein epitope provides a sufficient theoretical basis for p54 as a vaccine candidate protein. The present study uses a p54-specific nanobody as a probe to identify a highly conserved antigenic epitope, ^76^QQWVEV^81^, among different ASFV strains, and it can induce humoral immune responses in pigs. This is the first report using virus-specific nanobodies as a tool to identify some special epitopes that cannot be recognized by conventional monoclonal antibodies. This study opens up nanobodies as a new tool for identifying epitopes and also provides a theoretical basis for understanding p54-induced neutralizing antibodies.

## INTRODUCTION

African swine fever (ASF) is an acute, febrile, and highly contagious infectious disease in domestic pigs and wild boars, characterized by high morbidity and mortality; its causative agent is ASF virus (ASFV) (1). ASF outbreaks in major swine-raising countries in Europe, China, and some Southeast Asian countries, such as Thailand, Cambodia, and Vietnam, cause severe damage to the swine industry in affected countries, and it is thus listed as a notifiable infectious disease by the World Organisation for Animal Health (WOAH, formerly OIE) (2). Since the first outbreak of ASF in China’s Liaoning Province on 3 August 2018, as many as millions of pigs have been culled from pig farms across the country, and China classifies it as a class I zoonotic disease (3). At present, the development of vaccines and therapeutics against this specific virus has encountered great obstacles and challenges, and the disease remains the primary concern of major pig-raising countries worldwide (4).

ASFV belongs to the *Asfarviridae* family and *Asfivirus* genus; it is an enveloped double-stranded DNA virus with a genome size of about 170 to 193 kbp and is currently the only DNA arbovirus. The virus particles have an icosahedral structure (5). The protein composition and structure of ASFV are very complicated. According to related reports, the ASFV genome contains 150 to 167 open reading frames (ORFs) that encode more than 54 structural proteins and more than 100 nonstructural proteins, which are related to virus replication, structural morphology, infection, and induction of host anti-infection immunity (5, 6). The ASFV p54 protein, which is encoded by the *E183L* gene, is a major infectious protein and an essential component of virus survival and replication and is related to the adsorption of virus (7). p54 is a type II membrane-associated protein whose N terminus is located intracellularly and contains 60 amino acids (aa), followed by a transmembrane domain mainly located in the endoplasmic reticulum membrane and a 131-amino-acid C-terminal ectodomain; it elicits critical function in the transformation of viral envelope precursors (8, 9). Relevant predictions indicate that the extracellular domain of the p54 protein has multiple phosphorylation and glycosylation amino acid sites (10). p54 and p30 exert a synergistic role in ASFV binding to target cells (11). Some studies showed that ASFV p54 interacts with host dynein to facilitate viral transport to the cell perinuclear region after viral internalization into host cells (7). In addition, the p54 protein is able to induce neutralizing antibodies in pigs (11, 12). According to current immunological theory, the immunogenicity of most antigens is determined by the exposure of epitopes on the surface or specific domains of the antigens, which are the main target sites recognized by antibodies or immune cells. Identifying B cell epitopes and elucidating the humoral immune characteristics of viral proteins are of great significance for the development of new vaccines against ASFV. So far, anti-p54 monoclonal antibodies (MAbs) have been successfully produced, and several linear B cell epitopes were identified using these MAbs as an effective tool (13–15).

Domain antibody fragments of nanobodies (Nbs) are genetically engineered from heavy chain-only antibodies (HCAbs) present in camel peripheral blood and are naturally free of the heavy-chain first constant region (CH1) and light chain. Cloning and expression of variable heavy-chain domains are capable of making libraries of antibodies that bind different antigens, termed Nbs, also known as heavy-chain variable domains (VHHs), approximately 15 kDa in size. Nbs have longer CDR3 domains that can form more flexible paratopes targeting unconventional epitopes on cognate antigens (such as catalytic sites of enzymes), so they can recognize sites that are difficult for traditional antibodies (16, 17). Due to the presence of the extended antigen-binding domain, Nbs are able to bind to the corresponding targets with high affinity and specificity (18). Based on their advantages over traditional MAbs, Nbs have received more and more attention in the diagnosis and treatment of some major viral diseases, such as SARS-CoV-2, Middle East respiratory syndrome (MERS), etc. (19, 20). In fact, some research uses Nbs to identify special epitopes. For instance, Nbs against human proprotein convertase subtilisin/kexin 9 can recognize a linear epitope in the hinge domain that cannot be recognized by traditional MAbs (21). Due to the unique advantages of Nbs in recognizing antigenic epitopes, Nbs were used to identify epitope(s) of the p54 protein that were difficult to recognize by conventional antibodies in this study.

In our previous research, 13 Nbs (Nb8, Nb9, Nb10, Nb11, Nb13, Nb39, Nb45, Nb46, Nb56, Nb79, Nb81, Nb83, and Nb90) were screened from Bactrian camel immunized with a prokaryotic-expressed recombinant C-terminal domain (CTD) of the p54 protein (p54-CTD; aa 53 to 184 of the 184 aa) by using phage display technology (22). Using these screened Nbs as tools, epitope mapping of p54 polypeptide fragments and synthetic oligopeptides was performed. The results suggested that among the 13 Nbs, only Nb8 reacted strongly with the p54 protein, and finally, Nb8 was used as an antibody to identify the p54 epitope. Nb8-horseradish peroxidase (Nb8-HRP) reacted with specific linear amino acid epitope ranges from ^76^QQWVEV^81^ aa. Also, Nb8-HRP was able to react with the eukaryotic-expressed p54 protein and p54 of ASFV-infected cell samples, exhibiting good reactivity. The identified epitope ^76^QQWVEV^81^ was conserved among different ASFV strains in genotype II, and it could react with ASFV antibody-positive serums collected from naturally infected pigs, indicating that it is a natural linear epitope capable of eliciting humoral immune response during different ASFV strain infections. This is the first report on Nbs against the linear epitope of the ASFV p54 protein. These results provide biological materials and a molecular basis for basic and applied research on ASFV and provide a theoretical basis for the further development of Nbs into effective anti-ASFV therapeutic drugs or diagnosis reagent.

## RESULTS

### Production of 13 Nbs-HRP recombinant proteins against the ASFV p54 protein.

**FIG 1:**
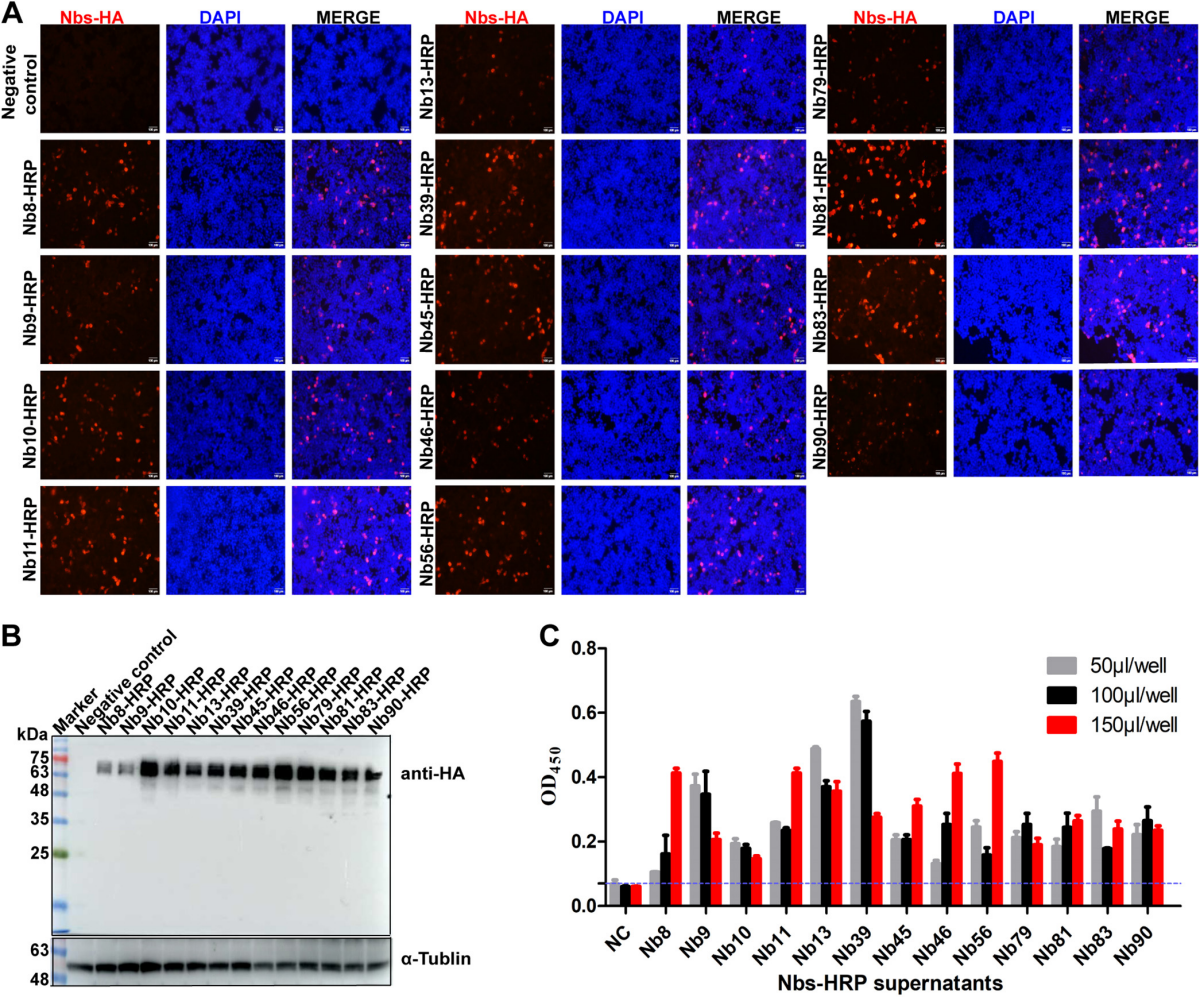
Expression and identification of 13 Nb-HRP proteins. pCAGGS-Nbs-HRP recombinant plasmids were transfected into HEK293T cells for 36 h. (A and B) Cells were harvested for Nb-HRP protein analysis using IFA (A) and Western blotting (B). (C) Cell culture supernatants were collected and serially diluted for secreted Nbs-HRP analysis by direct ELISA using p54-CTD (400 ng/well) as coating antigen.

The Nbs gene was amplified from the pCANTAB-5E vector and fused with the human Ig kappa (Hm Igκ) chain and HRP gene as previously reported (22) and then cloned into the pCAGGS-HA vector to construct pCAGGS-Nbs-HRP-HA recombinant plasmid. The sequences of the 13 Nbs were shown in our previous studies (22). IFA was performed to identify the expression of the 13 Nb-HRPs in HEK-293T cells. IFA results showed that all 13 Nb-HRPs were successfully expressed (Fig. 1A). Then, Western blot analysis was conducted to further confirm the expression of Nbs-HRP. Western blot results revealed that all 13 Nb-HRP fusion proteins were successfully expressed in HEK-293T cells with the expected size of 65 kDa (Fig. 1B). By carrying the Hm Igκ chain, the recombinant Nbs can be secreted into the cell culture supernatants (23). Direct ELISA results indicated that all the 13 Nb-HRP fusion proteins were successfully secreted into cell culture supernatants because the optical density at 450 nm (OD_450_) values of the tested samples were higher than that of the negative sample (Fig. 1C).

### Screening and characterization of anti-p54 Nb-HRP.

**FIG 2:**
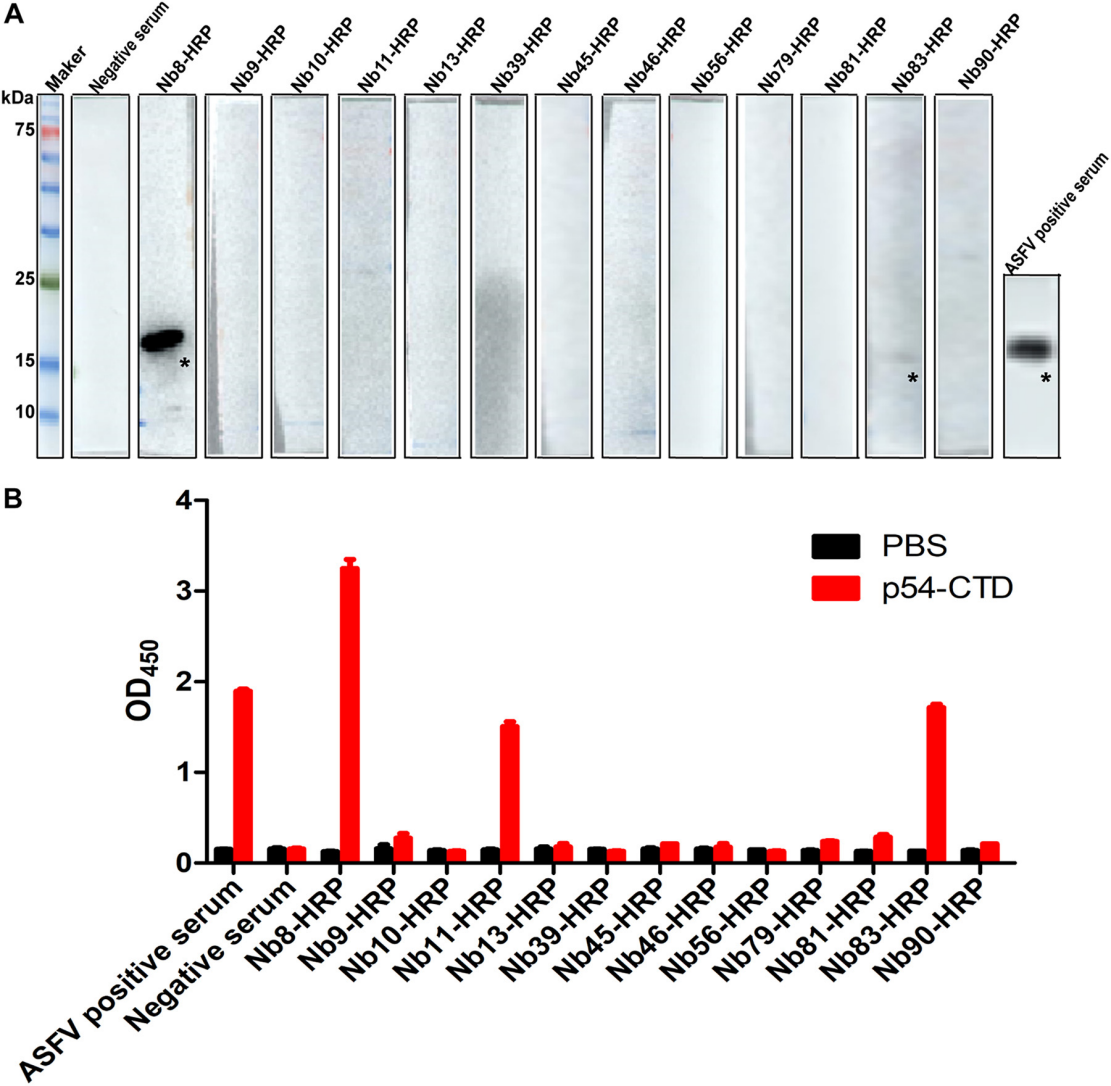
Reactivity of Nbs-HRP fusion protein with p54-CTD. (A) pET-30a-p54-CTD recombinant plasmids were transfected into E. coli BL21 expression competent cells and induced by IPTG (isopropyl-β-d-thiogalactopyranoside). Then, p54-CTD protein was purified and subjected to Western blot analysis using cell culture supernatants containing Nb8-, Nb9-, Nb10-, Nb11-, Nb13-, Nb39-, Nb45-, Nb46-, Nb56-, Nb79-, Nb81-, Nb83-, or Nb90-HRP as the enzyme-labeled antibody. (B) The purified p54-CTD protein was used as coating antigen (400 ng/well), and cell culture supernatants containing the 13 Nb-HRPs were used as the antibody to perform direct ELISA to analyze the reactivity of the 13 Nb-HRPs.

Nbs are able to recognize epitopes that cannot be recognized by conventional antibodies. To assess whether these Nbs could recognize some special epitopes different from those reported previously (13–15), Western blot analysis was performed to determine the reaction activity of the 13 Nbs with prokaryotic-expressed p54-CTD. Western blotting results showed that Nb8-HRP strongly reacted with prokaryotic-expressed p54-CTD, whereas Nb83-HRP supernatants reacted weakly, and the remaining 11 Nb-HRP supernatants did not react with p54-CTD absolutely (Fig. 2A). Direct ELISA results showed that p54-CTD reacted strongly with Nb8-HRP, weakly with Nb11- and Nb83-HRP supernatants, and could not react with the remaining 10 Nb-HRP supernatants (Fig. 2B). Therefore, Nb8-HRP was used for subsequent epitope identification and related experiments.

### Reactivity of Nb8 with ASFV-infected cells.

**FIG 3:**
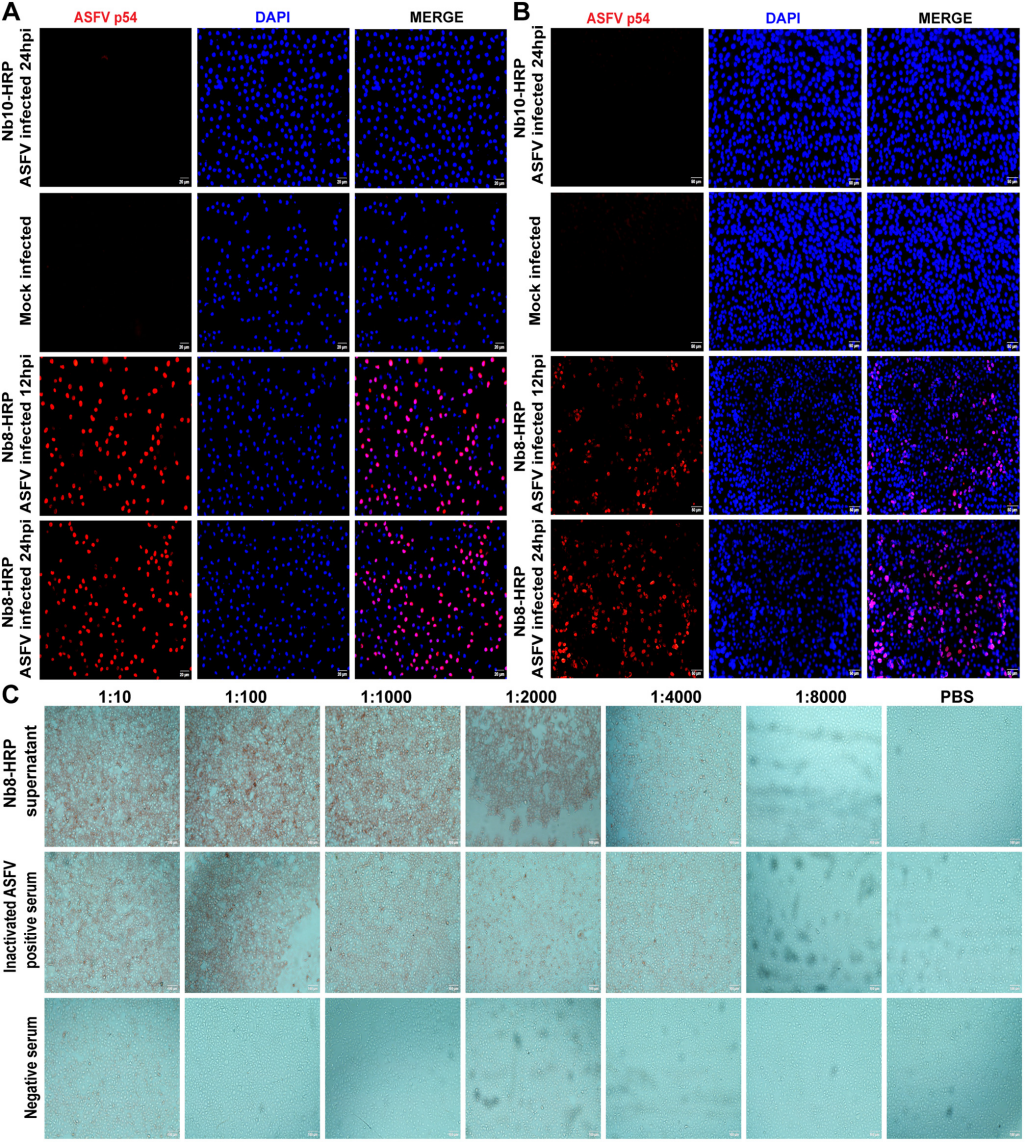
IFA and IPMA analysis of the Nb8-HRP reaction with the ASFV p54 protein. (A) IFA identification of the Nb8-HRP reaction with p54. (A and B) PAMs (A) or Vero cells (B) were mock infected or infected with the HLJ/18 ASFV strain at an MOI of 0.01 for 12 or 24 h. Then, cells were fixed and used for immunostaining analysis with Nb8-HRP as the first antibody, anti-HA MAb as the second antibody, and Alexa Fluor 594-conjugated IgG (H&L) as the third antibody. (C) IPMA identification of the Nb8-HRP reaction with p54. Vero cells were infected with the HLJ/18 ASFV strain at an MOI of 0.01 for 24 h. Cells were fixed and incubated with Nb8-HRP supernatants with a serial dilution of 1:10, 1:100, 1:1,000, 1:2,000, 1:4,000, or 1:8,000. Inactivated ASFV antibody-positive serums were diluted and used as the positive control, ASFV antibody-negative serums were used as the negative control, and PBS was used as the blank control.

The reactivity of Nb8 with ASFV-infected target cells was verified as well. IFA was performed on ASFV natural host cell porcine alveolar macrophages (PAMs). As shown in Fig. 3A, Nb8-HRP recognized ASFV-infected PAMs at both 12 and 24 hpi; nevertheless, the control supernatants from mock-infected or pCAGGS-Nb10-HRP-transfected cells did not react with ASFV-infected PAMs. The results were further confirmed in Vero cells infected with the ASFV HLJ/18 strain. Nb8 strongly labeled ASFV HLJ/18-infected Vero cells at both 12 and 24 hpi, while no fluorescence was observed in the Nb10-HRP-incubated or mock-infected cells (Fig. 3B). Taken together, these results suggest that Nb8-HRP could recognize ASFV HLJ/18-infected cells.

### Titration of Nb8 staining of infected Vero cells by IPMA.

Immunoperoxidase monolayer assay (IPMA) was performed to determine the reactivity of Nb8 with ASFV-infected Vero cells. As shown in Fig. 3C, both Nb8-HRP and inactivated ASFV antibody-positive serums were able to react with ASFV HLJ/18 strain-infected Vero cells. Nevertheless, neither pig-negative serum (negative control) nor phosphate-buffered saline (PBS) (blank control) was not able to react with ASFV HLJ/18 strain-infected Vero cells. The IPMA titers of Nb8-HRP were 1:4,000 (Fig. 3C, top), and the inactivated positive serum of recovered pigs was 1:4,000 as well (Fig. 3C, middle), while negative pig serum and PBS were not observed to react with virus.

### Epitope mapping of Nb8.

**FIG 4:**
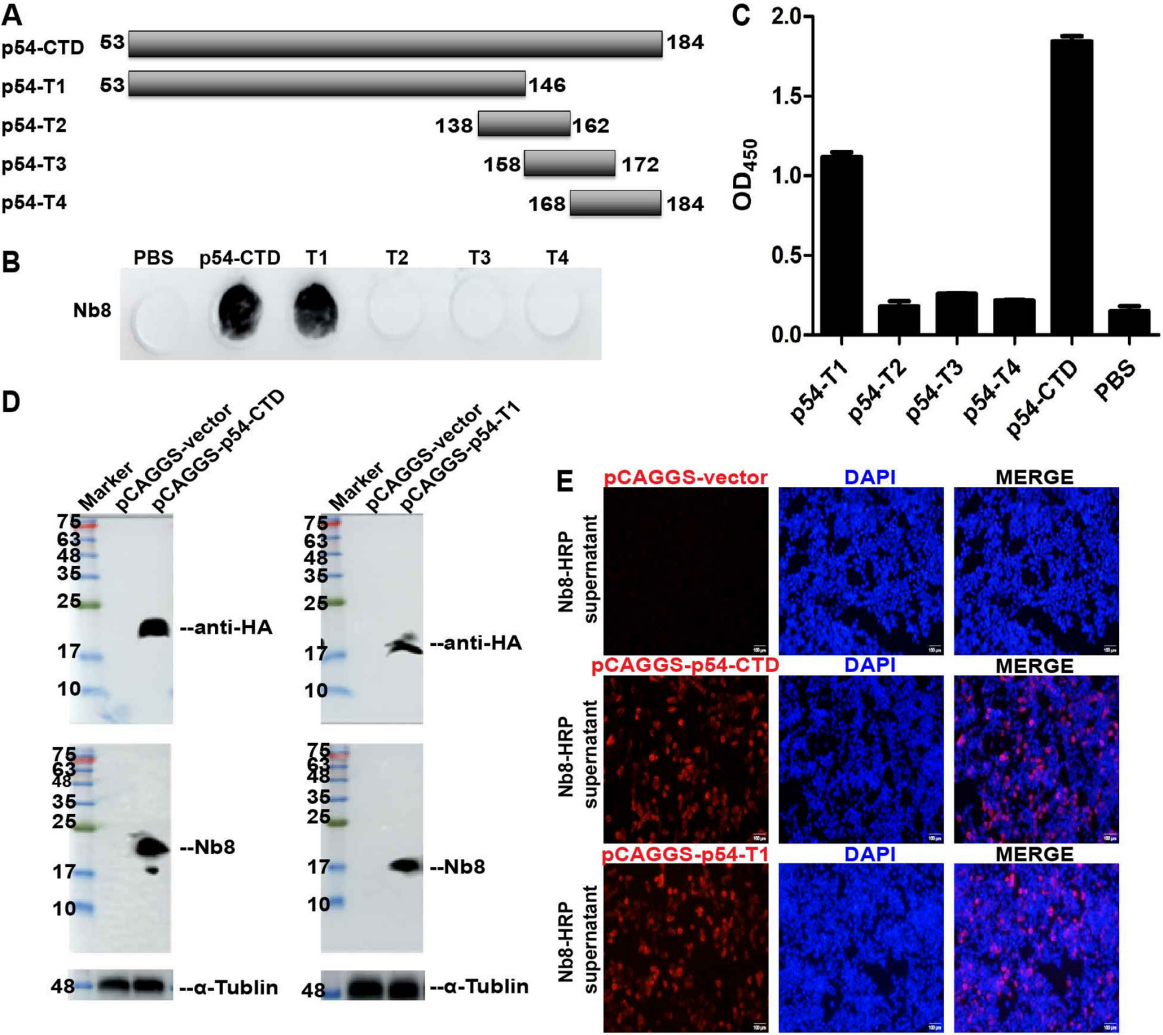
Reactivity of Nb8-HRP with p54 truncated mutants. (A) Schematic diagram of p54 truncated mutants and the synthetic polypeptides. (B and C) Dot blot (B) and peptide-based ELISA (C) analysis of Nb8-HRP’s reactivity with p54-CTD, p54-T1, and the synthetic polypeptides. pCAGGS-p54-CTD and pCAGGS-p54-T1 plasmids were transfected into HEK-293T cells for 36 h, respectively. The reactivity of Nb8-HRP with p54-CTD and p54-T1 were determined using Western blotting (D) and IFA (E), respectively. Cells were detected using anti-His MAb simultaneously as the control.

A schematic diagram of the eukaryotic expression of p54-CTD and one truncated mutant, p54-T1, and the mode of the synthesized polypeptides is shown in Fig. 4A. Dot blot results showed that in addition to p54-CTD, p54-T1 was also able to react with Nb8-HRP; however, the synthesized p54 polypeptides (p54-T2, -T3, and -T4) did not react with Nb8-HRP (Fig. 4B). Direct ELISA using the p54-CTD, -T1, -T2, -T3, and -T4 as coating antigen obtained similar results to the dot blot (Fig. 4C). p54-CTD was used as the positive control, and PBS was used as the negative control (in subsequent corresponding experiments, p54-CTD was used as a positive control, and PBS was used as a negative control, which will not be described in detail). Western blot results showed that both Nb8-HRP supernatants and anti-HA MAbs could react with p54-CTD and p54-T1 (Fig. 4D). Thus, the Nb8-HRP recombinant protein could recognize the epitope of p54-CTD located in p54-T1. IFA was conducted to further verify the Western blot results. IFA results also indicated that Nb8-HRP supernatants were able to react with p54-CTD and p54-T1 (Fig. 4E). Fluorescence was mainly distributed in the perinuclear region of pCAGGS-p54-CTD and -T1-transfected HEK-293T cells. Thus, p54-T1 was used for further epitope identification.

**FIG 5:**
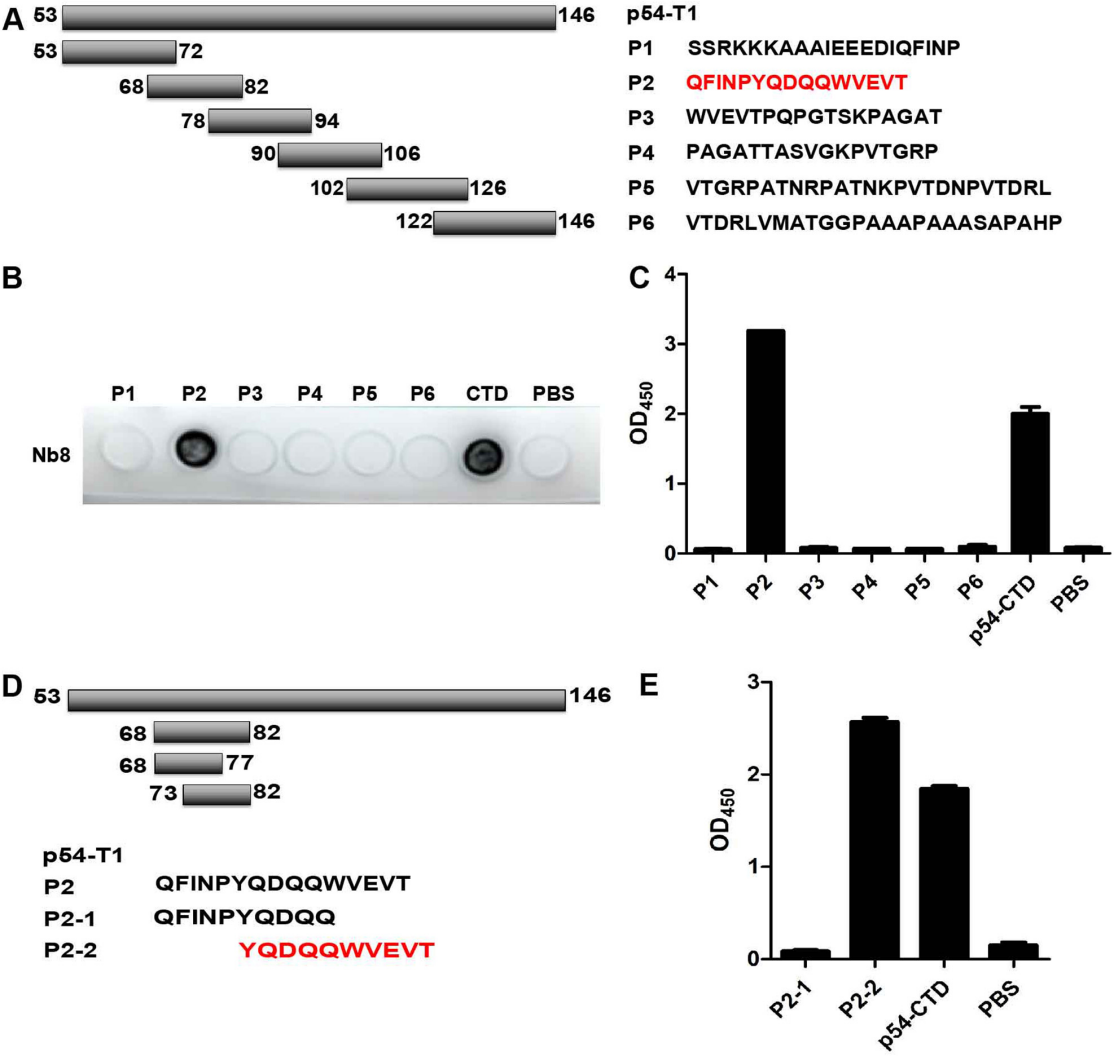
Location of the linear B cell epitope regions. (A) Schematic diagram of the synthesis of p54 polypeptides. (B and C) Dot blot (B) and peptide-based ELISA (C) analysis of Nb8-HRP reactivity with synthetic polypeptides. (D) Schematic diagram of further truncated peptides of p54-CTD. (E) Peptide-based ELISA analysis of Nb8-HRP reactivity with synthetic peptides. P1 to P6, 6 serial overlapping peptides covering the 53 to 146 aa of p54-CTD. P2-1 and P2-2, 2 serial overlapping peptides covering the 68 to 82 aa of p54-CTD.

To further clarify the recognition site of Nb8-HRP, six overlapping polypeptides (P1, P2, P3, P4, P5, and P6) covering the p54-T1 were synthesized (Fig. 5A). Then, dot blotting was performed to analyze which polypeptide could react with Nb8-HRP. Dot blot results showed that Nb8-HRP strongly bound to P2 (68 to 82 aa) (Fig. 5B), and peptide-based ELISA further corroborated the above-described results (Fig. 5C). To refine the motif recognized by Nb8-HRP, two truncations of P2 polypeptide were synthesized and detected by peptide-based ELISA (Fig. 5D). Peptide-based ELISA results suggested that Nb8-HRP could well recognize the polypeptide P2-2, but not P2-1 (Fig. 5E). Due to high hydrophobicity of the synthesized peptides, they were dissolved in dimethyl sulfoxide (DMSO), and dot blotting was not applicable for antigenic property analysis of P2-1 and P2-2.

### Core binding sites of Nb8 to the identified linear epitope.

**FIG 6:**
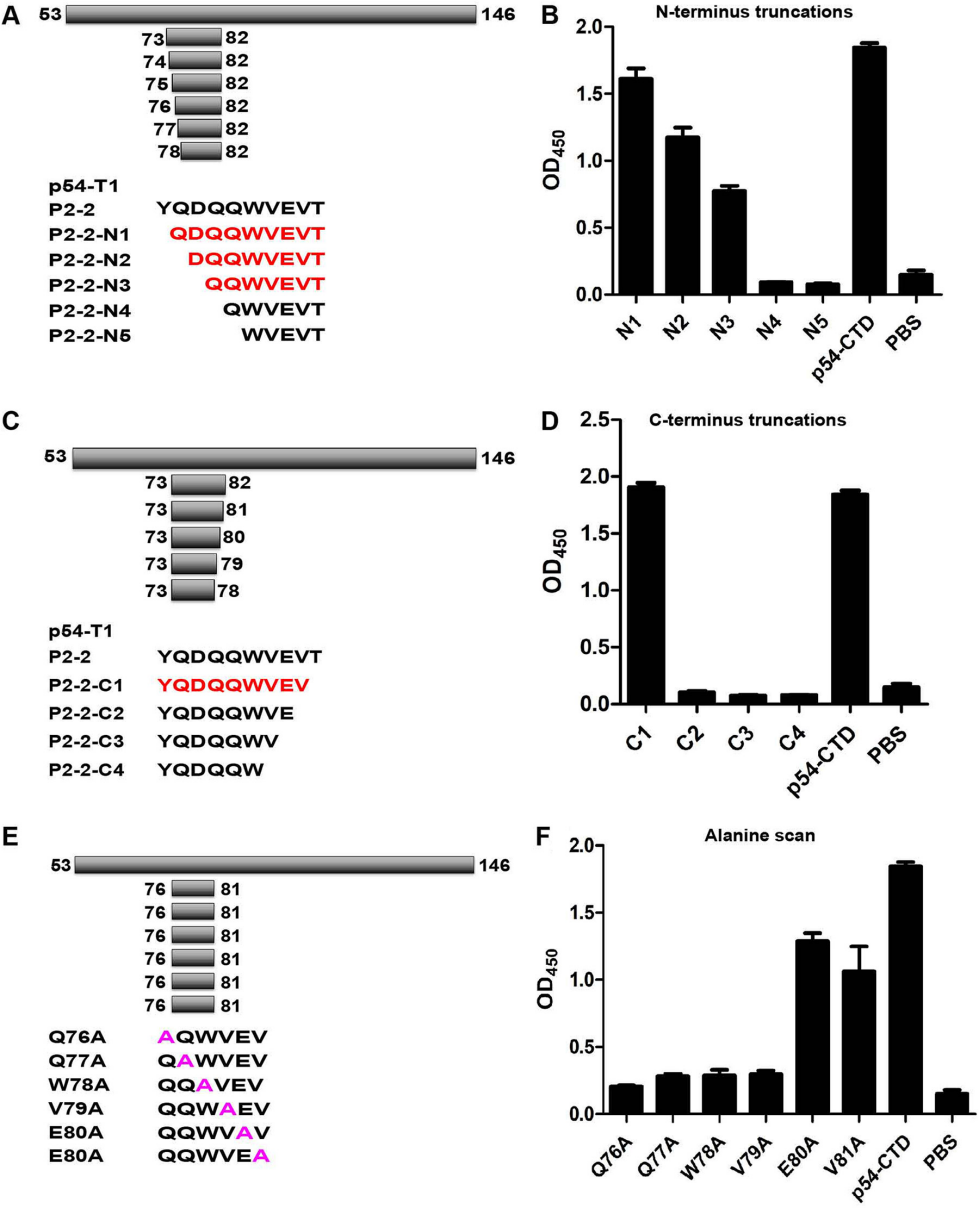
Mapping of critical binding sites recognized by Nb8. (A) Schematic diagram of N-terminal truncation. (B) Peptide-based ELISA analysis of Nb8-HRP critical binding sites. (C) Schematic diagram of C-terminal truncation. (D) Peptide-based ELISA analysis of Nb8-HRP critical binding sites. (E) Synthetic alanine mutant polypeptides. (F) Peptide-based ELISA analysis of Nb8-HRP core binding sites. N1 to N5, different truncated mutants from the N terminus; C1 to C4, different truncated mutants from the C terminus.

To further elucidate the core binding sites of Nb8 to the identified linear epitopes, a series of truncated peptides covering the N terminus of P2-2 were synthesized and used for peptide-based ELISA verification (Fig. 6A). The Nb8-HRP could recognize the peptides until deletion of amino acid residue ^73^YQDQ^76^ from the N terminus according to the results of peptide-based ELISA (Fig. 6B). The deletion of C-terminal residues of peptides obviously affects their recognition by Nb8-HRP since deletion of amino acid residues ^81^VT^82^ resulted in complete loss of ^73^YQDQVT^78^ binding to Nb8-HRP (Fig. 6C and D). As the synthetic amino acid peptides are highly hydrophobic, dot blot experiments were not applicable, and peptide-based ELISA was justly conducted. Taking the above-described results together, the peptide ^76^QQWVEV^81^ was the minimal linear epitope recognized by Nb8.

To identify the key sites for Nb8-HRP recognition, each amino acid residue of ^76^QQWVEV^81^ was sequentially substituted by alanine (Fig. 6E), and then, whether the alanine substitution affected its recognition by Nb8 was evaluated by peptide-based ELISA. The replacement of residue Q, Q, W, or V with an alanine residue (Q76A, Q77A, W78A, V79A) caused a marked decrease or complete loss of the antigenicity (Fig. 6F). However, the substitution of residue E or V with alanine residue (E80A, V81A) in epitope ^76^QQWVEV^81^ did not affect its reaction with Nb8-HRP (Fig. 6F), indicating that ^76^QQWV^79^ was the core binding site of Nb8-HRP. Dot blotting was not applicable due to the high hydrophobicity of the synthetic peptides.

### Reactivity of identified epitopes with inactivated ASFV antibody-positive serum.

**FIG 7:**
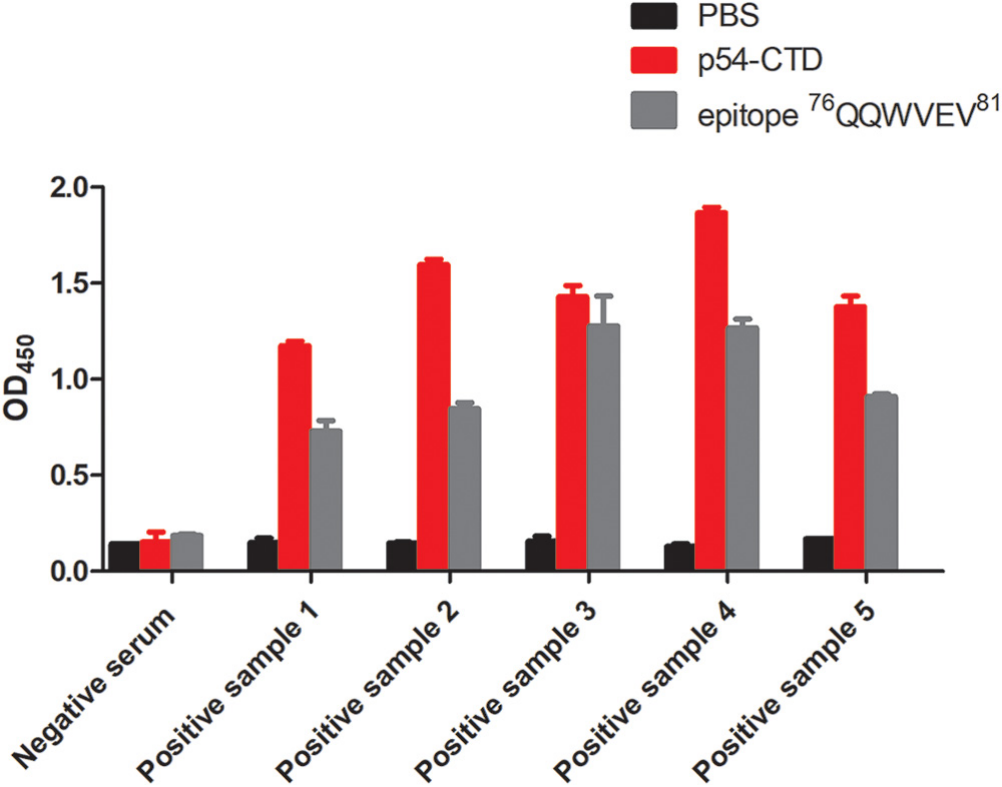
Reactivity of the identified epitopes with inactivated ASFV antibody-positive serum. Serum samples were obtained from 5 pigs naturally infected with ASFV. The reactivity of pig serums with identified epitope ^76^QQWVEV^81^ was determined by an absorbance value greater than 3 standard deviations above the absorbance for negative serum. Sample 1 to sample 5, serum from pigs naturally infected with ASFV.

To further verify whether the identified epitope ^76^QQWVEV^81^ could induce humoral immune responses in pigs, the reactivity of the epitope with inactivated ASFV antibody-positive serums collected from 5 different naturally infected pigs was determined using peptide-based ELISA. As expected, epitope ^76^QQWVEV^81^ reacted strongly with all the 5 inactivated ASFV antibody-positive pig serums (Fig. 7), suggesting that the identified epitope was most likely a natural linear B cell epitope that was capable of inducing a universal humoral immune response *in vivo*.

### Localization, characterization, and conservation analysis of the identified epitopes.

**FIG 8:**
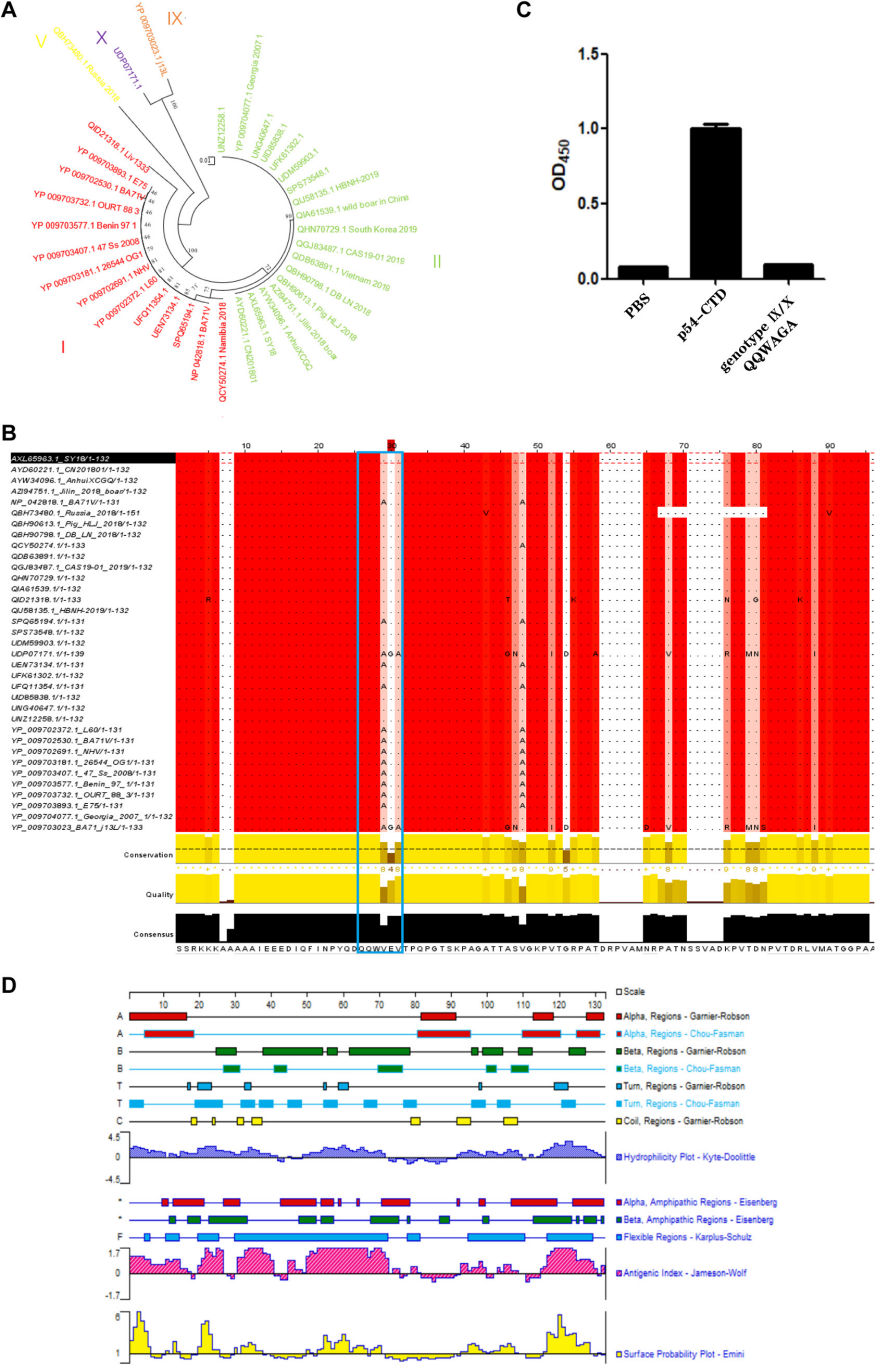
Bioinformatics analysis of the CTD antigenic regions among ASFV epidemic isolates. (A) Genotyping of 35 representative strains based on p54 protein sequences. The neighbor-joining (NJ) tree was reconstructed using MEGA 6.0. (B) Multiple-sequence alignment of the CTD of the p54 protein. The blue box indicates the CTD antigenic region, including epitope ^76^QQWVEV^81^. (C) Reactivity analysis of ^76^QWAGA^81^ from genotype IX and genotype X with Nb8-HRP by peptide-based ELISA. (D) Primary structure analysis of p54-CTD.

At present, there are 24 genotypes of ASFV that have been circulating in many pig-raising countries around the world. From the genotype perspective, all the ASFV strains isolated from Europe and Asia belong to genotypes I and II, while other strains, such as genotypes V, IX, and X, exist in Africa (24). Next, evolutionary relationship analysis was performed on the p54-CTD sequences of 35 different ASFV strains. Phylogenetic analysis showed that among the 35 ASFV strains, 14 belong to genotype I, 18 belong to genotype II, 1 belongs to type V, 1 belongs to type IX, and 1 belongs to type X. Further analysis showed that the ASFV strain currently circulating in Chinese pig farms is highly homologous to the Georgia 2007/1 strain, and both belong to genotype II (Fig. 8A).

Whether the identified epitope ^76^QQWVEV^81^ was conserved between diverse ASFV epidemic strains was analyzed. Eight reported Chinese epidemic isolates and 27 foreign isolates of ASFV were chosen for epitope analysis and comparison using Clustal Omega and Jalview software. The amino acid sequence alignment showed that epitope ^76^QQWVEV^81^ was highly conserved among various genotype II ASFV strains. In genotypes V, IX, and X, there were three substitutions (40% A at site 79, 5.7% G at site 80, and 5.7% A at site 81) of epitope ^76^QQWVEV^81^ (Fig. 8B). Based on the sequence alignment results, whether Nb8 could recognize antigen epitope at the same location of other ASFV strains, such as genotypes IX and X, was further determined. Peptide-based ELISA was used to further verify whether Nb8 recognizes ^76^QWAGA^81^ from genotype IX and genotype X. The results showed that Nb8-HRP could not react with ^76^QWAGA^81^ (Fig. 8C).

The epitope ^76^QQWVEV^81^ was analyzed for hydrophilicity and hydrophobicity, surface accessibility, protein flexibility, and antigenic index using DNASTAR Protean software. The secondary structure prediction results suggested that the identified epitope was located inside p54-CTD, with a high antigenic index and hydrophobicity (Fig. 8D). In addition, it may be a component of the α-helix and β-sheet (Fig. 8D), indicating that ^76^QQWVEV^81^ may be a pivotal B cell epitope on the p54 protein of the genotype II ASFV strain.

**FIG 9:**
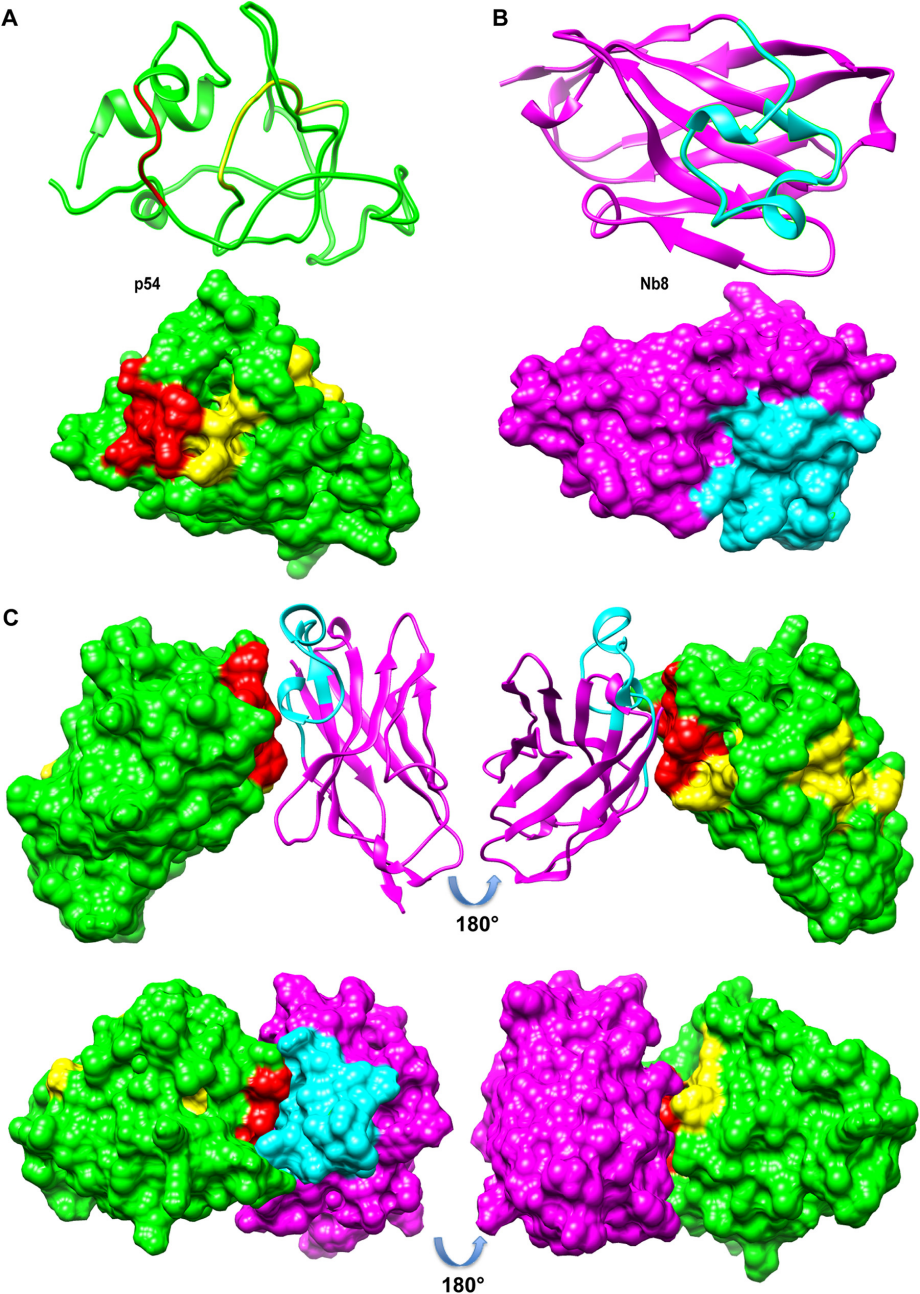
Localization of identified Nb8-binding epitope on the predicted 3D structural model of the ASFV p54-CTD protein. Secondary structural analysis and spatial distributions of the core epitope on the ASFV-p54-CTD (A), Nb8 (B), and the possible schematic diagram of p54-CTD-Nb8 complexes (C). The identified epitope, ^76^QQWVEV^81^, on p54 is marked in red, and near the C-terminal end is the dynein-binding domain (DBD) marked in yellow. The CDR3 domain of Nb8 is marked in blue.

The localization of identified Nb8-binding epitope on the predicted three-dimensional (3D) structural model of the ASFV p54-CTD protein was analyzed using the SWISS-MODEL online server. The predicted 3D models were drawn as cartoons and spheres for representation. The secondary structure of the ASFV p54-CTD protein was composed of a random coil, α-helix, and β-fold (Fig. 9A). The red callout stands for the identified epitope ^76^QQWVEV^81^ on p54, and the yellow callout, near the C-terminal end, stands for the dynein-binding domain (DBD) of p54 (Fig. 9A). The Nb8 protein structure contains multiple α-helices and β-sheets. The CDR3 domain of Nb8 is marked in blue (Fig. 9B). The 3D structural model of identified Nb8-binding epitope complex was predicted by ClusPro 2.0 (https://cluspro.bu.edu/), and the predicted results were analyzed by UCSF Chimera software. Localization of the identified Nb8-binding epitope ^76^QQWVEV^81^ on the predicted 3D model of ASFV p54-CTD protein is shown in Fig. 9C. Furthermore, the predicted ASFV p54-CTD and Nb8 3D structural model indicates that the CDR3 region of Nb8 may recognize the identified the epitope ^76^QQWVEV^81^ (Fig. 9C).

## DISCUSSION

African swine fever is an acute, highly pathogenic infectious disease that has caused huge economic losses to the world swine industry since it emerged from Africa in 1921 (25). ASF has become widespread in China’s pig farms, causing huge losses to China’s pig industry (26). So far, there are no effective vaccines or therapeutic drugs for ASF, which may be attributed to its complex virus structure and an immune mechanism that bring great difficulties to the development of new vaccines or drugs (27). Antigen epitopes play an important role in the induction of humoral immunity in pigs, and the identification of novel epitopes is helpful for an in-depth understanding of the characteristics of ASFV protein and also provides a reliable theoretical basis for the development of new vaccines.

Currently, ASFV proteins such as CD2v, p22, p30, p54, p72, D117L, pE199L, and pp62 are the main target proteins for the study and development of ASF subunit vaccines (28), but the immune effects produced by different protein combinations are often different, and most protein combinations cannot produce broad-spectrum cross-protection against different ASFV strains, suggesting that these proteins may not be enough for the development of ASF vaccines. Among the reported ASFV proteins, expression of p54, p30, and p72 proteins alone or in combination can induce neutralizing antibodies in pigs, and these antibodies can block ASFV adsorption, internalization, and other replication cycle processes and can also activate cytotoxic T lymphocyte responses (29). p54 is an important structural protein of ASFV, which plays an important role in the viral infection of host cells. Accurate epitope information is part of understanding and controlling the virus and informs the rational design of vaccines. Petrovan et al. used the p54 protein expressed in the baculovirus expression system to immunize mice, prepared a panel of MAbs, and identified 3 B cell epitopes, including 65 to 75 aa, 98 to 113 aa, and 118 to 127 aa (15).

In this study, we prepared 13 anti-p54 Nbs, used the overlapping peptide method to react the overlapping continuous peptides with the Nbs, and screened the peptide fragments that reacted with the Nbs, that is, the B cell epitope of the antigen. The linear epitope ^76^QQWVEV^81^ was identified finally, which has never been reported before, indicating that some hidden epitopes that are not easily recognized by traditional MAbs can be recognized by Nbs, showing the unique advantages of Nbs in identifying antigenic epitopes. Interestingly, of all the 13 Nbs, Nb11 and Nb83 hardly reacted with the p54 protein according to Western blot results (Fig. 2A), while both of them reacted with the p54 protein according to the direct ELISA results (Fig. 2B), this discrepancy may be due to higher sensitivity of ELISA than Western blotting, or Nb11 or Nb83 can recognize T cell epitopes in the p54 protein, which needs to be further verified. Also, our results indicate that Nb8-HRP can react with prokaryotic-expressed p54-CTD. Considering that the protein posttranslational modification system in eukaryotes is much more complex than that in prokaryotes, we continue to explore whether Nb8-HRP can recognize eukaryotic-expressed p54-CTD. The IFA results showed that Nb8-HRP can also react with eukaryotic expression of p54-CTD (Fig. 4E), indicating that the difference in modification ability between eukaryotic and prokaryotic organisms does not affect the recognition of antigen epitopes by Nb8 and further confirmed that Nb8 recognizes linear epitopes.

The identified epitope ^76^QQWVEV^81^ was highly conserved among genotype II strains, suggesting that Nb8 should be able to detect all genotype II ASFV strains. According to the IFA of ASFV-infected PAMs or IPMA results, Nb8 was able to recognize thep54 protein of ASFV-infected cells. Also, the epitope was able to react with naturally infected pig serums; thus, we speculate that the identified epitope occurs during ASFV infection *in vivo* and can induce serum antibodies. These results suggest that Nb8 is a good candidate as a competitive blocking antibody for genotype II ASFV infection. According to the sequence alignment results, amino acid residues V, E, and V of the identified epitope possess a high degree of variation among the genotypes II, V, IX, and X, which means that isolates within these genotypes would not be recognized by Nb8. This may also indicate that our previously developed competitive ELISA is not necessarily suitable for the detection of ASFV-specific serum antibodies of genotypes V, IX, and X (22).

In conclusion, a panel of anti-p54 Nbs was generated. Among these Nbs, Nb8 exhibits unique activities, which are briefly described as the following: (i) it reacted with ASFV-infected PAMs or Vero cells, (ii) Nbs can be viewed as an effective tool for identifying unconventional epitopes, and (iii) it recognized a new conserved epitope that could not be recognized by traditional MAbs. The specific Nb and Nb-based diagnostic assays would be important tools to aid in ASF disease control and prevention.

## MATERIALS AND METHODS

### Cells and inactivated ASFV antibody-positive serum.

HEK-293T and Vero cells, which were stored in our laboratory, were cultured at 37°C with 5% CO_2_ and maintained in Dulbecco’s modified Eagle’s medium (DMEM; Gibco, USA) supplemented with 10% fetal bovine serum (FBS; Gibco, Thermo Fisher Scientific, Inc.), 100 U/mL penicillin, and 100 μg/mL streptomycin (Life Technologies Corporation, Carlsbad, USA). The pig serums collected from pig farms were inactivated immediately after collection for 30 min at 60°C and identified as ASFV antibody positive using a commercial ELISA kit (Beijing Jinnuobaitai Biotechnology Co., Ltd.). ASFV antibody-negative serums were collected from officially certified ASFV pathogen-free pig farms.

### Production of 13 Nb-HRP recombinant proteins against p54-CTD.

In our previous research, a total of 13 specific Nbs against the ASFV p54-CTD protein were screened using phage display technology, and based on the features of the CDR3 region of the *VHH* gene, these Nbs were identified and named Nb8, Nb9, Nb10, Nb11, Nb13, Nb39, Nb45, Nb46, Nb56, Nb79, Nb81, Nb83, and Nb90, respectively (22). Following digestion with EcoRI and NheI, the Nb, human Ig kappa (Hm Igκ) chain, and horseradish peroxidase (HRP) fusion fragments were cloned into the pCAGGS-HA eukaryotic expression vector to construct pCAGGS-Nb-HRP recombinant plasmid (22). The correctly sequenced plasmids were transfected into HEK-293T cells using X-tremeGene HP DNA transfection reagent (Roche Diagnostics) to identify the expression and secretion of Nbs by Western blotting, indirect immunofluorescence assay (IFA), and direct ELISA.

For Western blotting of Nb expression, the transfected HEK-293T cells were collected at 48 h posttransfection (hpt) and lysed using NP-40 lysis buffer containing 1% protease inhibitor phenylmethanesulfonyl fluoride (Beyotime Institute of Biotechnology, Shanghai, China). Then, the samples were boiled at 95°C for 5 min and subjected to SDS-PAGE, transferred to polyvinylidene difluoride (PVDF) membrane, and probed with mouse anti-hemagglutinin (HA) monoclonal antibody (MAb, Beyotime Institute of Biotechnology) as the first antibody and HRP-conjugated goat anti-mouse IgG (H&L) (Beyotime Institute of Biotechnology) as the second antibody. The membranes were then visualized using enhanced chemiluminescent (ECL) reagents (Pierce, Rockford, IL, CA, USA).

For IFA analysis of Nbs expression, at 36 hpt, the HEK-293T cells were fixed with 4% paraformaldehyde at room temperature (RT) for 10 min, permeabilized with 0.3% Triton X-100 for 3 min, and blocked with 5% bovine serum albumin (BSA) for 1 h at RT. After washing 3 times using PBS, the cell samples were stained with mouse anti-HA MAb (Beyotime Institute of Biotechnology) and Alexa Fluor 594-conjugated goat anti-mouse IgG (H&L) (Abcam, Cambridge, MA, USA). Nuclei were stained with 4′,6-diamidino-2-phenylindole (DAPI). The fluorescent images were obtained using an inverted fluorescence microscope (Olympus Corporation, Tokyo, Japan).

For direct ELISA analysis of Nb secretion, the pCAGGS-Nbs-HRP-transfected HEK-293T cell culture supernatants at 48 hpt were harvested and coated into ELISA plates at volumes of 50, 100, and 150 μL/well at 4°C overnight. Then, the plates were washed 3 times using phosphate-buffered saline with Tween 20 (PBST) and followed by adding 100 μL of 3,3′,5,5′-tetramethylbenzidene (TMB) to each well and incubating in the dark for 15 min at 37°C. The reaction was stopped by adding 50 μL of 3 M H_2_SO_4_ to each well, and the absorbance of the samples at 450 nm was detected using a spectrophotometer (PerkinElmer, Inc.). According to the OD_450_ value, whether the Nbs-HRP recombinant protein was secreted into the cell culture supernatant was determined; 10% FBS plus DMEM was used as the control group during detection.

### Reactivity of Nb-HRPs with p54-CTD.

To better understand the characteristics of the ASFV p54 protein, the transmembrane region of p54 of the ASFV-China/2018/AnhuiXCGQ isolate (GenPept accession no. AYW34096) was predicted first using the DeepTMHMM website (https://dtu.biolib.com/DeepTMHMM). The results showed that its transmembrane region is located between 30 and 52 aa. Since proteins with a transmembrane domain are difficult to express, the transmembrane domain of p54 was deleted, and only the extracellular C-terminal domain (157 to 553 bp) was expressed. The coding DNA of the p54 C-terminal domain (p54-CTD) was synthesized and cloned into the pET-30a plasmid. After sequencing, the correct recombinant pET-30a-p54-CTD plasmids were transformed into Escherichia coli BL21(DE3) cells to express p54-CTD. To determine the reactivity of the screened 13 Nb-HRP supernatants with prokaryotic-expressed p54-CTD, Western blotting was performed using supernatants containing Nbs-HRP collected from pCAGGS-Nbs-HRP-transfected HEK-293T cells as the primary antibody (1:500 dilution).

Direct ELISA was performed to further analyze the reaction activity of Nbs-HRP with p54-CTD protein. The prokaryotic-expressed p54-CTD was coated into an ELISA plate (400 ng/well) at 4°C overnight. After blocking with 2.5% skim milk in PBST at 37°C for 1 h, 100-μL/well cell culture supernatants containing the 13 Nb-HRPs were added as the primary antibody and incubated for 1 h at 37°C. Then, the plates were washed with PBST 3 times and followed by adding 100 μL/well of TMB and incubated in the dark for 15 min at RT. We used 50 μL/well of 3 M H_2_SO_4_ to stop the reaction, and absorbance was measured at 450 nm on the spectrophotometer (PerkinElmer, Inc.). The reactivity of Nbs-HRP with p54-CTD was analyzed based on the OD_450_ value. We used 10% FBS plus DMEM as the control group simultaneously.

To determine whether Nb8-HRP reacted with eukaryotic expressed p54-CTD and its corresponding truncated mutant, p54-T1, both genes were cloned into pCAGGS-HA plasmid by primers pCAGGS-CTD-For and -Rev, pCAGGS-T1-For and -Rev (Table 1). After sequencing, the recombinant pCAGGS-p54-CTD and pCAGGS-p54-T1 were transfected into HEK-293T cells. At 48 hpt, the cells were harvested for Western blot analysis using Nb8-HRP supernatants as the primary antibody and then visualized directly using ECL reagents (Pierce).

**TABLE 1:** Primers used in this study

Primer	Sequence (5′–3′)^*a*^
pCAGGS-CTD-For	CCG**GAATTC**ATGCATATGCACCACCACCACCACCACT
pCAGGS-CTD-Rev	CCG**CTCGAG**TTAAAGCTTTCATTACAGGCTGTTCTCC
pCAGGS-T1-For	CCG**GAATTC**ATGCATATGCACCACCACCACCACCACT
pCAGGS-T1-Rev	CCG**CTCGAG**TTAGTGCGCCGGCGCGCTCGCCGCCGCC

a GAATTC, *Eco*R I recognition site; CTCGAG, *Xho* I recognition site.

For IFA analysis of reactivity of Nbs-HRP with p54-CTD, at 36 hpt, the HEK-293T cells were fixed using 4% paraformaldehyde at RT for 10 min, permeabilized with 0.3% Triton X-100 for 3 min, and blocked with 5% BSA for 1 h at RT, followed by washing 3 times using PBS and probed with Nb8-HRP supernatants as the primary antibody, mouse anti-His MAb as the second antibody (Beyotime Institute of Biotechnology; 1:1,000), and Alexa Fluor 594-conjugated goat anti-mouse IgG (H&L) as the third antibody (Abcam, 1:300). Cells were imaged under an inverted fluorescence microscope (Olympus Corporation).

### IPMA.

Vero cells were inoculated with the ASFV HLJ/18 strain at a multiplicity of infection (MOI) of 0.01, and at 24 hpi, the plates were inactivated. After blocking with 1% BSA at 37°C for 1 h and washing with PBST 3 times, supernatants containing the Nb8-HRP fusion protein, inactivated ASFV antibody-positive serum of ASFV-recovered pigs, and ASFV antibody-negative serum were diluted 1:10, 1:100, 1:1,000, 1:2,000, 1:4,000, and 1:8,000 and added into the plates as the primary antibodies, respectively (100 μL/well) and incubated at 37°C for 30 min. PBS was used simultaneously as a control. Followed by washing with PBST 3 times, the samples were incubated with or without HRP-conjugated goat anti-swine IgG (1:1,000; 100 μL/well) at 37°C for 30 min and then washed with PBST 3 times. Thirty microliters of 3-amino-9-ethylcazole (AEC) dye was added to each well and incubated at RT for 10 min, the residual AEC was washed away, double-distilled water (ddH_2_O) was added, and then the samples were observed and photographed using an inverted fluorescence microscope (Olympus Corporation). Cell samples of positive wells were brown-red, while cells of blank control wells and negative wells had no color when observed under the microscope. The cell samples were inactivated and kindly provided by Harbin Veterinary Research Institute and were stored at −20°C during shipping. All the live virus-related experiments were conducted in an animal biosafety level 3 lab (ABSL-3).

### IFA.

Vero cells or porcine alveolar macrophages (PAMs) were plated in 24-well cell culture plates 24 h prior to infecting with the ASFV HLJ/18 strain at an MOI of 0.01. At 12 and 24 hpi, the infected cells were fixed using 4% paraformaldehyde for 30 min at RT, permeabilized using 0.3% Triton X-100 for 3 min at RT, and then blocked using 5% BSA for 2 h at RT. Then, the samples were washed 3 times using PBS and followed by incubating with Nb8-HRP supernatants and Alexa Fluor-594 conjugated goat anti-mouse IgG (H&L) (Abcam). Nuclei were stained with DAPI, and the images were obtained using an inverted fluorescence microscope (Olympus Corporation). The ASFV-infected cell samples were inactivated and kindly provided by Harbin Veterinary Research Institute and were stored at −20°C during shipping.

### Design and synthesis of the p54 truncated peptide.

Based on in-depth analysis of amino acid sequence features of p54-CTD, a series of peptides to mimic the truncated fragment of ASFV p54 protein were further designed. Six overlapping peptides, which contained an offset of 5 aa and covered p54-T1 (aa 53 to 122), were synthesized to screen which could react with p54-specific Nbs. To precisely locate the epitope peptides capable of reacting with the specific Nbs, the identified peptide sequences were sequentially truncated from the N terminus and the C terminus, respectively. To further determine the key amino acid sites that decide the reaction of epitope with Nbs, alanine was used to replace the original amino acids in turn, and then dot blotting or direct ELISA was performed. All peptides used in the present experiments were synthesized by GenScript (Nanjing, China), and the purity was ≥95%.

### Dot blot assay.

For dot blot analysis of peptides reacted with specific Nb, 1 μg of each peptide was spotted onto nitrocellulose membranes (AE99; Schleicher & Schuell, Inc., Germany) until dry. Then, the membranes were blocked with 5% skim milk in PBST for 2 h at RT; after washing with PBST 3 times, the membranes were incubated with cell culture supernatants containing specific Nb-HRP as the primary antibody, with gentle shaking for 1 h at RT. After washing with PBST 3 times, the membrane was visualized using ECL reagent (Pierce).

### Peptide-based ELISA.

For peptide-based ELISA analysis of peptides reacted with specific Nbs, 400 ng/well of peptide dissolved in PBS (pH 7.4, 100 μL/well) was coated in 96-well ELISA plates at 4°C overnight. After washing with PBST 3 times, the plates were blocked with 5% skim milk in PBST for 1 h at 37°C. Then, Nb-HRP diluted with 5% skim milk in PBST was added and incubated at 37°C for 30 min, and then the plates were washed with PBST 3 times. One hundred microliters of TMB was added to each well for color reactions at 37°C for 15 min, and 50 μL/well of 3 M H_2_SO_4_ was used to stop the color reaction. The OD value of each well was measured at 450 nm using an ELISA microplate reader (PerkinElmer, Inc.).

### Biological information analysis.

Conservation of the identified epitope(s) in different ASFV reference strains was analyzed by comparing the identified epitope(s) of the p54 protein of the HLJ/18 strain with the other ASFV strains using the Clustal Omega (http://www.ebi.ac.uk/Tools/msa/clustalo/) and Jalview software. Based on the analysis data using SWISS-MODEL online server (https://swissmodel.expasy.org/), the 3D model of ASFV p54 and Nb8 was predicted using the UCSF Chimera software (https://www.cgl.ucsf.edu/chimera/). Analysis of spatial distribution and characterization of the identified epitopes of ASFV p54 protein were performed using Protean software (DNASTAR Inc., Madison, WI, USA).

### Ethics statement.

Animal experiments were approved by the Animal Care and Use Committee of Henan Agricultural University (ND19-12). We declare that the current research is in full compliance with ethical standards.
